# Parental educational status independently predicts the risk of prevalent hypertension in young adults

**DOI:** 10.1038/s41598-021-83205-0

**Published:** 2021-02-12

**Authors:** Sang Heon Suh, Su Hyun Song, Hong Sang Choi, Chang Seong Kim, Eun Hui Bae, Seong Kwon Ma, Soo Wan Kim

**Affiliations:** Department of Internal Medicine, Chonnam National University Medical School, 42, Jebong-ro, Dong-gu, Gwangju, 61469 Republic of Korea

**Keywords:** Cardiology, Health care, Medical research, Nephrology, Risk factors

## Abstract

Identification of individuals at risk of hypertension development based on socio-economic status have been inconclusive, due to variable definitions of low socio-economic status. We investigated whether educational status of individuals or their parents predicts prevalent hypertension in young adult population, by analyzing data of more than 37,000 non-institutionalized subjects from Korea National Health and Nutrition Examination Survey 2008 to 2017. Although low educational status of individual subjects was robustly associated with elevation of systolic blood pressure and increased prevalence of hypertension in general population, its impact on prevalent hypertension differed across age subgroups, and was remarkably attenuated in young adults. Parental educational status was significantly associated with prevalent hypertension in young adults, but not or only marginally in elderly population. Low parental educational status was also associated with high sodium intake in young adults, irrespective of subject’s own educational status. These collectively indicate that parental educational status, rather than individual’s own educational status, better and independently predicts prevalent hypertension in young adults, and that young adults with low parental educational status are prone to intake more sodium, possibly contributing to the increased risk of hypertension development. We expect that our findings could help define young individuals at risk of high sodium intake and hypertension.

## Introduction

Hypertension (HTN) is a potentially modifiable risk factor for cardiovascular mortality^1–3^, and is related to the progression of chronic illness, such as chronic kidney disease (CKD)^4^. Given the significant impact of HTN on the overall clinical outcomes, HTN per se has been a target of numerous interventional trials^5–7^. For a couple of decades, nevertheless, the incidence of HTN has been plateaued^2^, or, with a growing number of older individuals, is increasing even in the developed countries^8^. Moreover, the optimal management of HTN is compounded by several socio-economic conditions^2^, which is best illustrated by the young individuals with HTN^9^. The awareness of being hypertensive and the rate of blood pressure (BP) control in this population remain still poor, despite the remarkable improvement in the general population^2,8,9^. The disparity in health care delivery for HTN management, therefore, is yet to be overcome for such vulnerable populations.

Under-diagnosis of HTN in young subjects is an issue of particular medical importance. Previous cohort studies^10,11^ proved that HTN increases risk of cardiovascular and all-cause mortalities also in young adults. The diagnosis and management of HTN in young adults should also be distinct from those of the elderly, since the prevalence of secondary HTN is relatively high in this population^12^, although essential HTN is still the most common cause. Most importantly, the absence of evidence from randomized clinical trials on the benefit with pharmacologic interventions among the young hypertensive patients lead to a lack of recommendation for optimal BP management in this population^13^, emphasizing the primary prevention of HTN in young subjects with high risk.

Identification of young individuals at risk of HTN development based on socio-economic status (SES) has been previously reported^14–17^. Albeit the association of SES with HTN incidence has been suggested, a conclusive result has not been established yet, due to the lack of a consistent definition of SES that is variably assessed by educational status as well as income levels, marital status, and occupation of an individual^14–17^. Rather, since some of those factors are determined and only measurable later in adulthood, it is necessary that more simple and definitive criteria of high risk young individuals with HTN development should be presented. In this context, here we report low parental educational status as an independent risk factor of prevalent HTN in young adults, which is an essentially simple and pre-determined socio-economic condition. By analyzing a nation-wide survey data from more than 37,000 non-institutionalized subjects, we demonstrated that parental educational status better predicts the prevalence of HTN than the subject’s own educational status, especially in young adults.

## Results

### Baseline characteristics of study subjects by educational status

To address the baseline characteristics according to educational status, study subjects were divided by educational years into 4 subgroups (Table 1). The mean age was significantly younger in the subjects with longer education years. The frequency of female subjects decreased as the educational duration increased. Parental educational status was significantly associated with education status of the study participants; in subjects with educational duration ≤ 6 years, more than 90 % of parents were educated for ≤ 6 years, while only less than 1 % of parents were educated for > 12 years. In subjects with educational duration > 12 years, 27.7% of parents were educated for ≤ 6 years, whereas 23.1% of parents were educated for > 12 years, suggesting that the educational attainments of parents and their offspring are roughly proportionate. The ratio of urban residence significantly increased with the educational duration of individual subjects. Urinary sodium excretion (Na^+^/Cr) in random urine sample and estimated 24-h urine sodium was inversely related with subject's educational duration. SBP, DBP, waist circumference (WC) and body mass index (BMI) were also inversely correlated with subject’s educational duration. Estimated glomerular filtration rate (eGFR) were more preserved in subjects with longer educational years, although no clear trend was observed between the prevalence of proteinuria and the subject’s educational status. The prevalence of co-morbid conditions, such as HTN, diabetes mellitus (DM), dyslipidemia, coronary artery disease, stroke, anemia, and history of smoking, was significantly higher in subjects with lower educational status. Collectively, these suggests that the educational status of individuals is closely related with their medical conditions.

**Table 1:** Baseline characteristics of study subjects by educational status.

	Education years				
	≤ 6	7–9	10–12	> 12	
Numbers	9129	4211	12,564	12,088	
Age (years)	66.668 ± 9.275	58.076 ± 10.636	44.667 ± 15.153	41.872 ± 12.516	< 0.001
Female (%)	6289 (68.9)	2240 (53.2)	6509 (51.8)	5802 (48.08)	< 0.001
Urban residence (%)	3695 (40.5)	2121 (50.4)	6706 (53.4)	7258 (60.0)	< 0.001
**Parental education years ≤ 12 (%)**					< 0.001
≤ 6	6804 (91.1)	2947 (78.6)	5466 (45.5)	3305 (27.7)	
7–9	353 (4.7)	428 (11.4)	2219 (17.3)	2064 (17.3)	
10–12	256 (3.4)	297 (7.9)	2824 (23.5)	3816 (31.9)	
> 12	56 (0.7)	78 (2.1)	1507 (12.5)	2764 (23.1)	
**Urine chemistry**					
Random urine Na^+^ (mEq/L)	126.672 ± 48.230	126.535 ± 49.690	121.151 ± 51.852	115.550 ± 50.988	< 0.001
Random urine Cr (mg/dL)	115.360 ± 64.727	130.784 ± 69.874	159.626 ± 89.487	168.258 ± 87.148	< 0.001
Random urine Na^+^/Cr	1.453 ± 0.958	1.233 ± 0.783	0.994 ± 0.660	0.864 ± 0.563	< 0.001
Estimated 24-h urine Na^+^ (g)	8.589 ± 2.193	8.562 ± 2.061	8.092 ± 2.055	7.814 ± 1.965	< 0.001
Systolic blood pressure (mmHg)	127.753 ± 17.585	122.437 ± 16.921	116.106 ± 15.872	112.963 ± 14.519	< 0.001
Diastolic blood pressure (mmHg)	75.117 ± 10.208	76.474 ± 10.251	75.300 ± 10.319	74.931 ± 10.281	< 0.001
Waist circumference (cm)	84.168 ± 9.253	83.502 ± 9.238	80.944 ± 10.058	80.412 ± 10.282	< 0.001
Body mass index (kg/m^2^)	24.264 ± 3.335	24.193 ± 3.182	23.677 ± 3.488	23.447 ± 3.440	< 0.001
eGFR (mL/min/1.73 m^2^)	81.187 ± 16.219	87.510 ± 16.014	96.252 ± 18.006	96.547 ± 16.835	< 0.001
Urine protein ≥ 1+ (%)	800 (8.8)	338 (8.0)	1172 (9.3)	976 (8.1)	0.002
**Co-morbidities**					
Hypertension (%)	4993 (54.8)	1637 (38.9)	2863 (22.8)	2040 (16.9)	< 0.001
Diabetes (%)	1786 (20.2)	662 (16.1)	1071 (8.7)	650 (5.5)	< 0.001
Dyslipidemia (%)	1941 (38.9)	817 (34.0)	1358 (19.8)	987 (13.7)	< 0.001
Coronary artery disease (%)	470 (10.4)	163 (7.5)	203 (3.1)	136 (2.0)	< 0.001
Stroke (%)	415 (9.2)	137 (6.4)	167 (2.6)	70 (1.0)	< 0.001
Anemia (%)	1049 (11.6)	330 (7.9)	1051 (8.4)	843 (7.0)	< 0.001
History of smoking (%)	2819 (31.2)	1846 (44.0)	5309 (42.4)	4912 (40.7)	< 0.001

Values for categorical variables are given as number (percentage); values for continuous variables, as mean ± standard deviation. P value by one-way analysis of variance and χ^2^ test for continuous and categorical variables, respectively. GFR, estimated glomerular filtration rate.

### The impact of educational status on prevalent HTN differs across age subgroups

To better characterize the impact of educational status on BP and prevalent HTN, since the gap in the mean age of subjects with the lowest and highest educational attainments was more than 20 years, the subjects were further stratified by their ages; 19–39 years, 40–59 years, and 60–80 years (Supplementary Table S1 online). The comparison of systolic blood pressure (SBP), diastolic blood pressure (DBP), and prevalent HTN in each subgroup revealed that SBP increased as educational duration decreased, regardless of age (Supplementary Table S2 online). DBP also peaked in subjects with education duration ≤ 6 years, except but in subgroups with age 60–80 years, where DBP peaked in subjects with educational duration 10–12 years. However, in subjects with age 19–39 years, the prevalence of HTN did not significantly differ by educational status, while in subjects with age 40–59 and age 60–80 years, prevalence of HTN were inversely correlated with educational status.

To figure out whether educational status was independently associated with SBP and prevalent HTN, a series of regression models were analyzed. The analyses of entire subjects revealed that low educational status significantly increases SBP and prevalence of HTN, even after adjustment of co-variates (Supplementary Tables S3 and S4 online). Similarly, in the analyses of subgroups stratified by age, low educational status was independently associated with increased SBP in all subgroups (Table 2). Conversely, the prevalence of HTN in subjects with age 19–39 years was not significantly associated with their educational status, even though low educational status independently increased the prevalence of HTN in subgroups with age 40–59 and 60–80 years. The analysis of a restricted cubic spline model with adjustment of co-variates demonstrated the overall impact of an individual’s own educational status becomes more evident as the age increases (Figure S2). Taken together, despite the robust association in general population, the impact of educational status on prevalent HTN differed across age subgroups, and was remarkably attenuated in young adults.

**Table 2:** Impact of low educational status on SBP and prevalent HTN in the subgroups stratified by age.

SBP	Model 1		Model 2		Model 3		Model 4	
	Coefficients (95%CIs)	*P* value	Coefficients (95%CIs)	*P* value	Coefficients (95%CIs)	*P* value	Coefficients (95%CIs)	*P* value
Age, 19–39 years	1.385 (0.727, 2.042)	< 0.001	1.114 (0.483, 1.746)	0.001	0.803 (0.194, 1.413)	0.010	0.830 (0.219, 1.440)	0.008
Age, 40–59 years	3.845 (3.128, 4.562)	< 0.001	2.954 (2.224, 3.685)	< 0.001	2.438 (1.723, 3.152)	< 0.001	2.400 (1.681, 3.118)	< 0.001
Age, 60–80 years	3.698 (2.414, 4.981)	< 0.001	2.198 (0.890, 3.489)	0.001	2.003 (0.699, 3.307)	0.003	1.915 (0.611, 3.220)	0.004

Prevalent HTN	Odds ratio (95%CI)	*P* value	Odds ratio (95%CI)	*P* value	Odds ratio (95%CI)	*P* value	Odds ratio (95%CI)	*P* value
Age, 19–39 years	0.923 (0.789, 1.080)	0.317	1.112 (0.944, 1.311)	0.205	1.021 (0.792, 1.316)	0.874	1.033 (0.801, 1.332)	0.805
Age, 40–59 years	1.457 (1.343, 1.581)	< 0.001	1.304 (1.195, 1.424)	< 0.001	1.261 (1.112, 1.431)	< 0.001	1.263 (1.112, 1.433)	< 0.001
Age, 60–80 years	1.346 (1.197, 1.514)	< 0.001	1.177 (1.042, 1.330)	0.009	1.200 (1.018, 1.413)	0.029	1.199 (1.017, 1.413)	0.030

Model 1, unadjusted. Model 2, adjusted for age and sex. Model 3, model 2 + adjusted for co-morbidities (high body mass index, high waist circumference, diabetes, dyslipidemia, coronary artery disease, stroke, and history of smoking). Model 4, model 3 + adjusted for eGFR and proteinuria).

CI, confidence interval; HTN, hypertension; SBP, systolic blood pressure.

### Parental educational status independently predicts prevalent HTN in young adults

Pursuing a socio-economic factor that predicts the risk of prevalent HTN in young adults, we focused on the role of parental educational status (Supplementary Table S5 online), as we hypothesized that the parental educational status might be critical for the socio-economic environment during childhood and juvenile periods of the subject, contributing to the formation of health behavior thereafter. In contrast to the educational status of study subjects, the educational status of parents significantly altered SBP, DBP, and prevalence of HTN in all age subgroups (Table 3). To validate independent associations of parental educational status with SBP and prevalent HTN, co-variates including the educational status of study subjects were adjusted in regression analyses, which revealed that low parental educational status was not independently associated either with SBP or with prevalent HTN (Supplementary Tables S6 and S7 online). Intriguingly, the association between low parental educational status and prevalent HTN was seen before adjustment with educational status of study subjects, but turned to be not significant after adjustment with the co-variate. As the impact of educational status on prevalent HTN differed across age subgroups (Table 2), the subgroups were analyzed to test age-specific impact of parental educational attainment on SBP or prevalent HTN (Table 4), where parental educational status was independently associated with both SBP and prevalent HTN in the subjects with age 19–39 years. Parental educational status was not independently associated with SBP in analyses of subjects with age 40–59 and age 60–80 years. The association of parental educational status and prevalent HTN was not significant in the analysis of subjects with age 40–59 years, and was only marginally significant (*P* = 0.049) in the analysis of subjects with age 60–80 years. To summarize, low parental educational status independently predicted elevated SBP and prevalent HTN specifically in young adults, although its association with SPB or prevalent HTN was much weaker in general population. No significant association between low parental educational status and SBP was observed in the subgroups stratified by a number of variables, such as sex, WC, BMI, and history of DM, dyslipidemia, stroke, or CKD (Table 5).

**Table 3:** Comparison of SBP, DBP, and prevalent HTN according to parental educational status in subgroups stratified by age.

	Parental education (years)				
	≤ 6	7–9	10–12	> 12	*P* value
**Age, 19–39 years**					
SBP (mmHg)	109.904 ± 12.339	109.572 ± 12.548	109.592 ± 12.127	108.427 ± 11.362^b,c,e^	
DBP (mmHg)	73.244 ± 10.118	72.946 ± 10.533	72.905 ± 9.946	72.068 ± 9.124^b,c,e^	
Prevalent HTN	147 (7.6)	138 (7.4)	259 (6.2)	117 (4.5)	
**Age, 40–59 years**					
SBP (mmHg)	118.652 ± 16.105	116.15.866^b^	115.586 ± 15.154^b^	115.016 ± 15.154^b,d^	< 0.001
DBP (mmHg)	77.967 ± 10.286	77.803 ± 10.467	77.420 ± 10.361	77.301 ± 10.549	0.042
Prevalent HTN	2166 (28.0)	553 (23.2)	494 (21.2)	316 (23.7)	< 0.001^f^
**Age, 60–80 years**					
SBP (mmHg)	128.013 ± 17.452	126.776 ± 16.391	126.345 ± 16.217	125.739 ± 17.832^a^	0.002
DBP (mmHg)	74.332 ± 9.980	74.784 ± 9.337	75.242 ± 9.892	75.069 ± 9.082	0.044
Prevalent HTN	5041 (57.0)	446 (55.5)	376 (57.6)	215 (48.0)	0.002^f^

Values for categorical variables are given as number (percentage); values for continuous variables, as mean ± standard deviation. ^a^*P* < 0.05, ^b^*P* < 0.001 *vs.* subjects with parental education year ≤ 6; ^c^*P* < 0.05; ^d^*P* < 0.01 *vs.* subjects with parental education year 7–9; ^e^*P* < 0.01 *vs.* subjects with parental education year 10–12 by one-Way ANOVA with Tukey's multiple comparison test. ^f^*P* value by Pearson Chi-square test.

DBP, diastolic blood pressure; HTN, hypertension; SBP, systolic blood pressure.

**Table 4:** Impact of low parental educational status on SBP and prevalent HTN in the subgroups stratified by age.

SBP	Model 1		Model 2		Model 3		Model 4		Model 5	
	Coefficients (95%CIs)	*P* value	Coefficients (95%CIs)	*P* value	Coefficients (95%CIs)	*P* value	Coefficients (95%CIs)	*P* value	Coefficients (95%CIs)	*P* value
Age, 19–39 years	1.363 (0.645, 2.081)	< 0.001	1.205 (0.540, 1.870)	< 0.001	0.861 (0.223, 1.498)	0.008	0.863 (0.225, 1.500)	0.008	0.803 (0.164, 1.442)	0.014
Age, 40–59 years	2.433 (1.273, 3.593)	< 0.001	1.468 (0.344, 2.591)	0.010	1.017 (− 0.074, 2.108)	0.068	0.993 (− 0.098, 2.085)	0.074	0.131 (− 0.994, 1.255)	0.820
Age, 60–80 years	2.875 (0.844, 4.906)	< 0.001	1.906 (− 0.106, 3.918)	0.063	1.746 (− 0.265, 3.757)	0.089	1.730 (− 0.279, 3.740)	0.091	0.990 (− 1.091, 3.071)	0.351

Prevalent HTN	Odds ratio (95%CI)	*P* value	Odds ratio (95%CI)	*P* value	Odds ratio (95%CI)	*P* value	Odds ratio (95%CI)	*P* value	Odds ratio (95%CI)	*P* value
Age, 19–39 years	1.558 (1.269, 1.912)	< 0.001	1.261 (1.019, 1.560)	0.033	1.427 (1.054, 1.934)	0.022	1.436 (1.060, 1.946)	0.019	1.442 (1.064, 1.956)	0.018
Age, 40–59 years	1.123 (0.983, 1.282)	0.087	0.980 (0.855, 1.124)	0.776	0.928 (0.765, 1.125)	0.445	0.851 (0.697, 1.039)	0.113	0.851 (0.697, 1.039)	0.113
Age, 60–80 years	1.434 (1.186, 1.733)	< 0.001	1.278 (1.055, 1.549)	0.012	1.369 (1.061, 1.767)	0.016	1.370 (1.06, 1.769)	0.16	1.304 (1.001, 1.699)	0.049

Model 1, unadjusted. Model 2, adjusted for age and sex. Model 3, model 2 + adjusted for co− morbidities (high body mass index, high waist circumference, diabetes, dyslipidemia, coronary artery disease, stroke, and history of smoking). Model 4, model 3 + adjusted for eGFR and proteinuria). Model 5, model 4 + adjusted for educational status of individual subjects.

CI, confidence interval; HTN, hypertension; SBP, systolic blood pressure.

**Table 5:** Impact of low parental educational status and SBP in various subgroups stratified by other than age.

	Coefficients (95%CI)	*P* value
**Sex**		
Male	0.271 (− 0.655, 1.197)	0.566
Female	− 0.244 (− 1.120, 0.631)	0.584
**WC**		
≥ 90 cm for male, ≥ 80 cm for female	− 0.275 (− 1.010, 0.461)	0.464
< 90 cm for male, < 80 cm for female	0.069 (− 1.185, 1.322)	0.914
**BMI**		
≥ 25 kg/m^2^	− 0.394 (− 1.146, 0.358)	0.304
< 25 kg/m^2^	0.272 (− 0.934, 1.478)	0.658
**History of diabetes**		
Yes	2.865 (− 0.019, 5.749)	0.052
No	− 0.211 (− 0.866, 0.443)	0.527
**History of dyslipidemia**		
Yes	1.979 (− 0.216, 4.175)	0.077
No	− 0.138 (− 0.805, 0.529)	0.684
**History of stroke**		
Yes	3.147 (− 6.543, 12.836)	0.523
No	0.013 (− 0.630, 0.655)	0.969
**History of CKD**		
Yes	1.453 (− 0.739, 3.644)	0.194
No	− 0.056 (− 0.724, 0.613)	0.870

Models were adjusted for age, sex, co-morbidities (high body mass index, high waist circumference, diabetes, dyslipidemia, coronary artery disease, stroke, and history of smoking), estimated glomerular filtration rate, proteinuria, and educational status of individual subjects.

BMI, body mass index; CI, confidence interval; CKD, chronic kidney disease; WC, waist circumference.

### Low parental educational status is associated with high sodium intake in young adults

**Figure 1:**
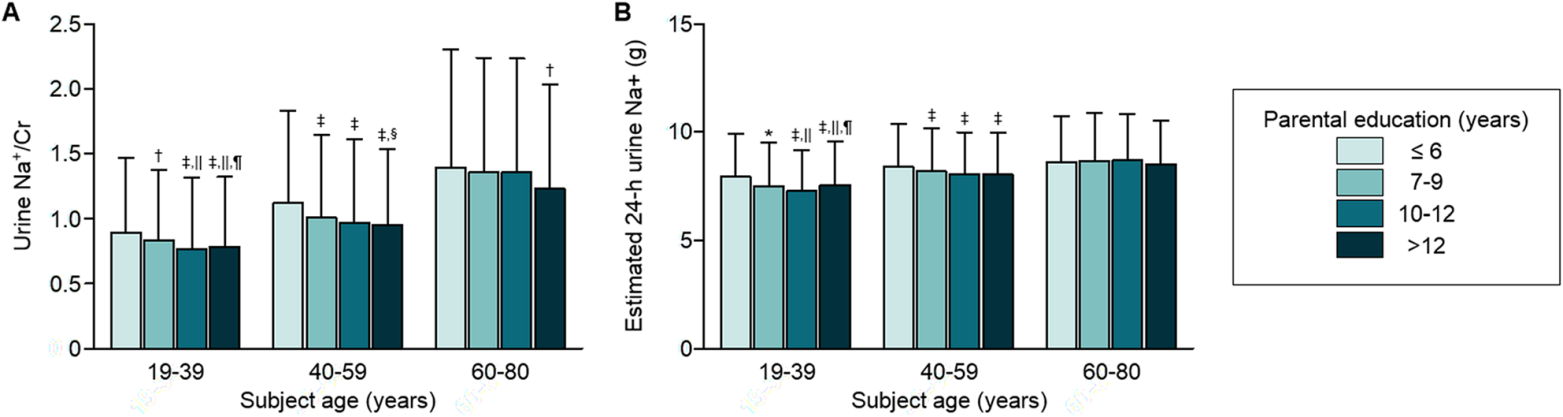
Comparison of urine sodium excretion by educational attainment and age. Error bars mean standard deviation. *P < 0.05, ^†^*P* < 0.01, ^‡^*P* < 0.001 *vs.* subjects with parental education year ≤ 6; ^§^*P* < 0.05, ^||^*P* < 0.001 *vs.* subjects with parental education year 7–9; ^¶^*P* < 0.001 *vs.* subjects with parental education year 10–12 by one-Way ANOVA with Tukey's multiple comparison test.

To unveil the mechanism linking the parental educational status and prevalent HTN in young adults, we compared urine sodium excretion of the subjects (Fig. 1), as evidences so far indicate an essential role of excess sodium intake in the development of HTN^5–7,18,19^. Na^+^/Cr in random urine and estimated 24-h urine sodium increased as the subject ages increase. Na^+^/Cr in random urine and estimated 24-h urine sodium significantly differ according to parental educational status in the subjects with age 19–39 years and, to a less degree, in the subjects with age 40–59 years, which finding was remarkably blunted in subjects with age 60–80 years. Although the association between low parental educational attainment and random urine Na^+^/Cr was significant before adjustment with educational status of study subjects, but was no more valid after adjustment with the co-variate in the regression analysis of the entire study subjects (Supplementary Table S8 online), while subgroup analyses demonstrated that parental educational status was independently associated with increased random urine Na^+^/Cr in subjects with age 19–39 years, irrespective of subject’s own educational status, but not in subjects with age 40–59 or with age 60–80 years (Table 6). Therefore, these suggest that low parental educational status is associated with sodium intake in young adults, possibly contributing to the increased risk of HTN development.

**Table 6:** Impact of low parental educational status on random urine Na^+^/Cr in the subgroups stratified by age.

	Model 1		Model 2		Model 3		Model 4		Model 5	
	Coefficients (95%CIs)	*P* value	Coefficients (95%CIs)	*P* value	Coefficients (95%CIs)	*P* value	Coefficients (95%CIs)	*P* value	Coefficients (95%CIs)	*P* value
Age, 19–39 years	0.647 (0.623, 0.671)	< 0.001	0.036 (0.007, 0.065)	< 0.001	0.035 (0.006, 0.065)	0.018	0.033 (0.005, 0.062)	0.021	0.030 (0.001, 0.058)	0.042
Age, 40–59 years	0.088 (0.044, 0.132)	< 0.001	0.052 (0.010, 0.095)	0.016	0.050 (0.007, 0.092)	0.022	0.040 (− 0.002, 0.082)	0.059	0.010 (− 0.033, 0.053)	0.652
Age, 60–80 years	0.135 (0.036, 0.234)	0.008	0.110 (0.014, 0.207)	0.029	0.108 (0.011, 0.205)	0.029	0.107 (0.012, 0.203)	0.027	0.088 (− 0.011, 0.187)	0.082

Model 1, unadjusted. Model 2, adjusted for age and sex. Model 3, model 2 + adjusted for co-morbidities (high body mass index, high waist circumference, diabetes, dyslipidemia, coronary artery disease, stroke, and history of smoking). Model 4, model 3 + adjusted for eGFR and proteinuria). Model 5, model 4 + adjusted for educational status of individual subjects. CI, confidence interval.

## Discussion

In the present study, we discovered that low parental educational status predicts prevalent HTN in young adults, but not in the middle-aged and elderly population. Although educational status of individual subjects is robustly associated with increases SBP and prevalence of HTN in general population, its impact on prevalent HTN differs across age subgroups, and is remarkably attenuated in young adults. Low parental educational status is also associated with high sodium intake in young adults, irrespective of subject’s own educational status, possibly contributing to the increased risk of HTN development.

Of noticeable finding in this study is that the association between low parental attainment and HTN (Supplementary Table S7 online) or between low parental attainment and high dietary sodium intake (Supplementary Table S8 online) is only significant before adjustment with subject’s own educational status in general population, suggesting that the impact of parental educational attainment on HTN and dietary sodium intake is substantially overcome by educational status of individual subjects. The analyses of subgroups stratified by ages also revealed that the effect of parental educational status on BP (Table 3) and dietary sodium intake (Table 4) was much clearly observed in the young adults, and was no more or only marginally valid in the subgroups with age > 40. These are in line with the previous studies of HTN in childhood and adolescent periods, where elevated BP was consistently associated with parental educational attainment^20–22^, but not with grandparental educational attainment^23,24^ in this population, collectively suggesting that, as the age increases, the role of parental education status as a ‘socio-economic legacy’ fade away, and that the educational status of individual subjects emerges to dominantly determine lifestyle and health behavior.

An observational study including 498 adolescent participants previously reported that the effect of parental education on BMI, lipid profiles, and SBP during young adulthood was statistically significant, but was no more significant after adjusting for participants’ own education^25^. The result may seem slightly contradictory to our findings, since the cross-sectional analyses of more than 37,000 participants in the current study demonstrated that low educational attainment of parents is still a risk factor for high salt intake and HTN in young adults even after adjusting for educational status of individual study subjects. We, however, also observed that, with aging, the role of the individual’s own educational status predominates in HTN prevalence and dietary sodium intake. (Tables 3 and 4). Therefore, findings both from the previous and current studies commonly emphasize that educated individuals would be less likely to develop HTN.

The mechanism how educational status is biologically transduced into HTN development is still elusive. A previous study indicated the BP in early life of the subjects with low parental educational attainment might track into adulthood^26^, while the rationale for elevated BP during childhood and adolescent period is lacking. Another study proved that the educational status of the young individual subjects overcomes the impact of parental educational attainment on BP^25^, although the biological explanation to link parental educational status and HTN development was not presented. In this regard, we hypothesized the contribution of sodium intake to HTN development in relation to educational status of individual subjects and their parents. Indeed, it has been believed that high sodium intake is closely linked to HTN development. Experimental evidences demonstrated that high dietary salt inhibits normal function of vascular endothelial cells to reduce nitric oxide synthesis and promote arterial stiffness^18^, leading to the elevation of BP. Cross sectional analysis of clinical data revealed that the excess amount of daily sodium intake is associated with poor BP control rate^19^. Most importantly, interventional studies to restrict dietary sodium intake alone or along with body weight reduction have proved the benefits in SBP and DBP control^5–7^. We demonstrated that random urine Na^+^/Cr is significantly associated with parental educational status specifically in young adults (Table 4), proving the hypothesis that high sodium intake of the young individuals with low parental educational status contributes to HTN development. Nevertheless, our results do not exclude the possible role of other factors in the development of HTN of individuals with low parental educational status, and the precise mechanism should be further clarified to more delicately guide the prevention of HTN in the vulnerable population.

In conclusion, we report that parental educational status, rather than individual’s own educational status, better and independently predicts prevalent HTN in young adults, and that young adults with low parental educational status are prone to intake more sodium, possibly contributing to the increased risk of HTN development. Particular concerns are required for young hypertensive subjects, as the delivery optimal medical care is compounded in this population. We, therefore, expect that our findings could help define young individuals at risk of high sodium intake and HTN development.

## Methods

### Study design and participants

The Korea National Health and Nutrition Examination Survey (KNHANES) is a nationwide population-based cross-sectional study of the health and nutritional status of the noninstitutionalized Korean population. It consists of a health questionnaire, physical/laboratory examinations, and nutrition survey. The present study analyzed data obtained from KNHANES 2008 to 2017 (Figure S1 in Supplemental Data), because the measurement of urine sodium and creatinine has been included in the data since 2008. Written informed consent was obtained from each participant in KNHANES at the time of enrollment. Of 85,036 participants in the health questionnaire and physical/laboratory examination of KNHANES 2008 to 2017, those who are less than 19 years old, pregnant, or lacking one of the following information were excluded (n = 47,044): SBP and DBP, urine sodium and creatinine measurement, serum creatinine measurement, dip stick urine protein in subjects with eGFR ≥ 60 mL/min/1.73 m^2^, or educational attainment of the subject and parents. Finally, 37,992 participants were included in analyses. This study protocol was approved by the Institutional Review Board of Chonnam National University Hospital (CNUH-EXP-2019-294), and was conducted in accordance with the Declaration of Helsinki and its later amendments or comparable ethical standards.

### Anthropometric and laboratory data

Trained medical staff performed physical examinations following standardized procedures. BP was measured manually 3 times at 30-s intervals after a minimum of 5 min of rest in a seated position and recorded as the average value of the 2nd and 3rd measurements. Blood samples were collected after at least an 8-h fast, properly processed, immediately refrigerated, and transported in cold storage to the central laboratory (Neodin Medical Institute, Seoul, Korea) within 24 h. eGFR was calculated from serum creatinine level using the CKD-Epidemiology Collaboration equation^27^. Urine sodium and creatinine concentration were determined in random urine specimen. Proteinuria was defined as albuminuria (≥  1+) determined by dipstick urine test.

### Demographic and clinical characteristics

Educational status was dichotomized into high and low both in the subjects and in their parents, where education year > 12 was considered as high^20^, and education year ≤ 12 was considered as relatively low. The highest among paternal and maternal educational attainment was determined as parental educational attainment. Smoking was dichotomized as current/former smoker or non-smokers. BMI ≥ 25 kg/m^2^ was defined as high. WC ≥ 90 cm in men or ≥ 80 cm in women was defined as high. HTN was defined as SBP  ≥ 140 mmHg, DBP ≥ 90 mm Hg, or use of anti-hypertensive medication. DM was defined as serum fasting glucose level ≥ 126 mg/dL, use of antidiabetic medicine, or a physician diagnosis of diabetes mellitus. Anemia was defined as hemoglobin level < 13 g/dL for men and < 12 g/dL for women. History of coronary artery disease including angina pectoris and acute myocardial infarction, stroke, and dyslipidemia was defined either by self-report or physician diagnosis. CKD was defined as the presence of proteinuria or eGFR < 60 mL/min/1.73 m^2^^28^.

### Estimation of daily sodium intake from random urine specimen

To evaluate daily sodium intake, instead 24-h urine collection^29,30^, 24-h urinary sodium excretion was estimated from the sodium and creatinine of random urine samples according to the following equation^31^: 24-h urinary Na^+^ excretion (mEq/day) = 21.98 $\times$ U_Na_/U_Cr_ $\times$ [–2.04 $\times$ Age + 14.89 $\times$ Weight (kg) + 16.14 $\times$ Height (cm) – 2244.45]^0.392^, where U_Na_ and U_Cr_ indicate sodium concentration (mEq/L) and creatinine concentration (mg/dl) in the spot urine, respectively. Since the correlation with 24-h urinary sodium excretion has been validated^32^, the ratio of sodium to creatinine (Na^+^/Cr) in random urine specimen was also calculated.

### Statistical analysis

Data are presented as the mean ± standard deviation for continuous variables, and as number, or percent for categorical variables. To compare the difference in the baseline characteristics according to educational status of individuals and their parents, one-way analysis of variance and χ^2^ test were used for continuous and categorical variables, respectively. The association between educational attainments of the subjects or their parents and BP, prevalence of HTN, or urinary sodium excretion was investigated by multivariate logistic regression methods adjusting for indicated variables in each table. A restricted cubic spline model with adjustment of indicated variables was analyzed to delineate the association between age and the risk of HTN by the individual subject’s educational status. Statistical analyses were performed with SPSS (version 20.0; SPSS Inc). *P* < 0.05 was considered statistically significant.

## Supplementary Information

Supplementary Information.
