# Supplementary material for: Parental educational status independently predicts the risk of prevalent hypertension in young adults
Source: Sci Rep. 2021 Feb 12;11:3698. doi: 10.1038/s41598-021-83205-0 (PMC7881088; doi:10.1038/s41598-021-83205-0)
Supplement: Supplementary file 1 — Supplementary Information. [file 41598_2021_83205_MOESM1_ESM.pdf]

Supplementary Information

Parental educational status independently predicts the risk of prevalent hypertension in young adults

Sang Heon Suh, Su Hyun Song, Hong Sang Choi, Chang Seong Kim, Eun Hui Bae, Seong Kwon Ma, and Soo Wan Kim

Department of Internal Medicine, Chonnam National University Medical School, Gwangju, Korea

Figure S1. Flow chart of study participants.

Figure S2. Association between age and the risk of HTN by the individual subject's educational status

Table S1. Baseline characteristics of the subgroups stratified by age

Table S2. Comparison of SBP, DBP, and prevalent HTN according to educational status in subgroups stratified by age

Table S3. Association between low educational status and SBP

Table S4. Association between low educational status and prevalent HTN

Table S5. Baseline characteristics of study subjects by parental educational status

Table S6. Association between parental educational attainment and SBP

Table S7. Association between parental educational attainment and prevalent HTN

Table S8. Association between parental educational status and random urine Na^+/Cr

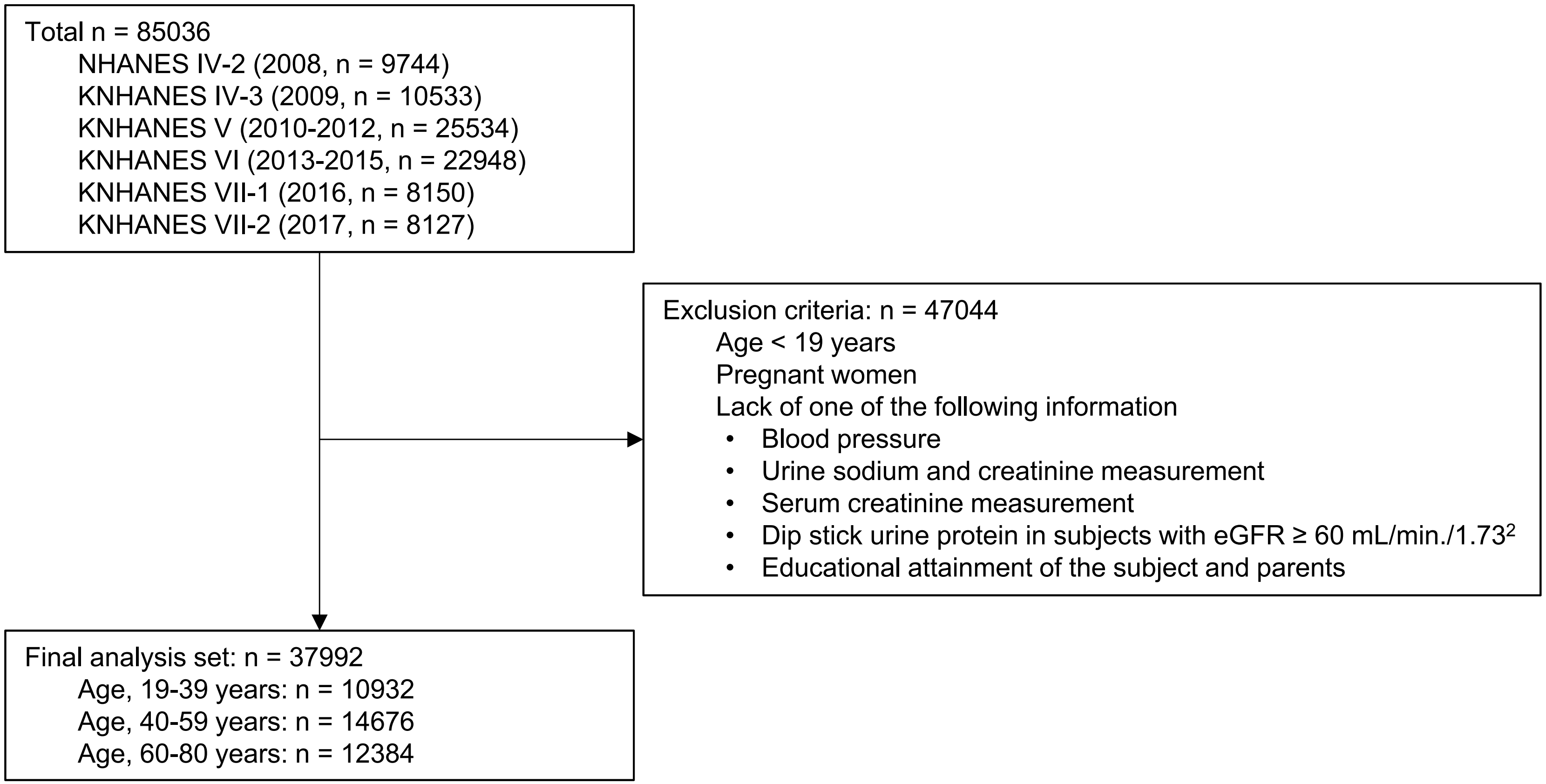

Figure S1. Flow chart of study participants.

Abbreviation: eGFR, estimated glomerular filtration rate; KNHANES, Korea National Health and Nutrition Examination Survey.

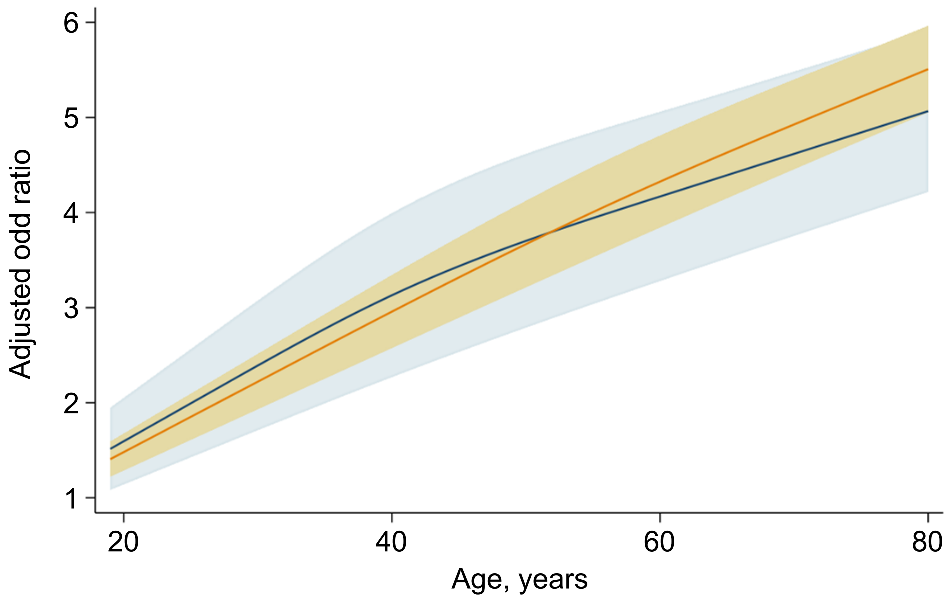

Figure S2. Association between age and the risk of HTN by the individual subject's educational status.

Restricted cubic spline curve illustrating the association between age and the risk of HTN by the individual subject's educational status. The model was adjusted for sex, co-morbidities (high body mass index, high waist circumference, diabetes, dyslipidemia, coronary artery disease, stroke, and history of smoking), estimated glomerular filtration rate, proteinuria. Orange line, low educational status; Blue line, relatively high educational status. The shaded area represents the 95% confidence interval.

Table S1. Baseline characteristics of the subgroups stratified by age

	Age subgroup (years)			
	19-39	40-59	60-80	*P* value
Numbers	10932	14676	12384	
Age (years)	30.617 ± 5.942	49.664 ± 5.765	69.198 ± 6.094	< 0.001
Female (%)	5803 (53.1)	8207 (55.9)	6830 (55.2)	< 0.001
Urban residence (%)	6228 (57.0)	7746 (52.8)	5806 (46.9)	< 0.001
Education years (%)				< 0.001
≤ 6	71 (0.6)	1954 (13.3)	7104 (57.4)	
7-9	196 (1.8)	2151 (14.7)	1864 (15.1)	
10-12	4670 (42.7)	5717 (39.0)	2177 (17.6)	
> 12	5995 (54.8)	4854 (33.1)	1239 (10.0)	
Parental education years ≤ 12 (%)				< 0.001
≤ 6	1938 (18.2)	7735 (56.1)	8849 (82.3)	
7-9	1876 (17.6)	2384 (17.3)	804 (7.5)	
10-12	4205 (39.5)	2335 (16.9)	653 (6.1)	
> 12	2621 (24.6)	1336 (9.7)	448 (4.2)	
Urine chemistry				
Na^+ (mEq/L)	118.333 ± 54.227	121.043 ± 50.555	124.200 ± 47.345	< 0.001
Cr (mg/dL)	190.287 ± 96.122	143.712 ± 76.321	117.408 ± 64.140	< 0.001
Na^+/Cr	0.788 ± 0.540	1.067 ± 0.682	1.383 ± 0.908	< 0.001
Estimated 24-h urine Na^+ (g)	7.574 ± 2.008	8.266 ± 1.991	8.598 ± 2.142	< 0.001
Systolic blood pressure (mmHg)	109.403 ± 12.101	117.641 ± 15.890	127.874 ± 17.334	< 0.001
Diastolic blood pressure (mmHg)	72.790 ± 9.899	77.826 ± 10.330	74.427 ± 9.876	< 0.001
Waist circumference (cm)	78.533 ± 10.773	82.032 ± 9.351	84.508 ± 9.100	< 0.001
Body mass index (kg/m^2)	23.134 ± 3.812	24.069 ± 3.241	24.075 ± 3.172	< 0.001
eGFR (mL/min./1.73m^2)	106.496 ± 14.556	92.416 ± 13.919	77.965 ± 14.796	< 0.001
Urine protein ≥ 1+ (%)	1085 (9.9)	1105 (7.5)	1096 (8.9)	< 0.001
Co-morbidities				
Hypertension (%)	679 (6.2)	3808 (26.0)	7046 (57.0)	< 0.001
Diabetes (%)	188 (1.8)	1315 (9.2)	2666 (22.1)	< 0.001
Dyslipidemia (%)	213 (3.9)	1889 (22.5)	3001 (39.1)	< 0.001
Coronary artery disease (%)	5 (0.1)	193 (2.5)	774 (11.0)	< 0.001
Stroke (%)	9 (0.2)	159 (2.1)	621 (8.9)	< 0.001
Anemia (%)	765 (7.0)	1115 (7.6)	1393 (11.3)	< 0.001
Smoking (%)	4168 (38.2)	5801 (39.6)	4917 (40.0)	0.01

Note: Note: Values for categorical variables are given as number (percentage); values for continuous variables, as mean ± standard deviation. P value by one-way analysis of variance and $\chi 2$ test for continuous and categorical variables, respectively. Abbreviation: eGFR, estimated glomerular filtration rate.

Table S2. Comparison of SBP, DBP, and prevalent HTN according to educational status in subgroups stratified by age

	Education (years)				*P* value
	≤ 6	7-9	10-12	> 12	
Age, 19-39 years					
Systolic blood pressure (mmHg)	113.634±10.917	110.837±13.510	109.982±12.123	108.855±12.016[a, f]	< 0.001
Diastolic blood pressure (mmHg)	75.493±9.378	74.015±10.717	72.630±9.825	72.842±9.929	0.021
Incident hypertension (%)	8 (11.3)	15 (7.7)	271 (5.8)	385 (6.4)	0.133[g]
Age, 40-59 years					
Systolic blood pressure (mmHg)	122.749±16.654	120.260±16.348[b]	117.072±15.817[b, d]	115.095±14.779[b, d, f]	< 0.001
Diastolic blood pressure (mmHg)	78.676±10.133	78.266±10.242	77.581±10.338[b, c]	77.577±10.415[b, c]	< 0.001
Incident of hypertension (%)	718 (36.8)	641 (29.8)	1416 (24.8)	1033 (21.3)	< 0.001[g]
Age, 60-80 years					
Systolic blood pressure (mmHg)	129.270±17.571	126.170±16.909[b]	126.704±16.932[b]	124.489±16.444[b, c, e]	< 0.001
Diastolic blood pressure (mmHg)	74.135±10.018	74.666±9.837	75.035±9.896a	74.673±8.983	0.001
Incident hypertension (%)	4267 (60.1)	981 (52.6)	1176 (54.0)	622 (50.3)	< 0.001[g]

Note: Values for categorical variables are given as number (percentage); values for continuous variables, as mean ± standard deviation. [a] $P < 0.01$, [b] $P < 0.001$ *vs.* subjects with parental education year ≤ 6; [c] $P < 0.05$; [d] $P < 0.001$ *vs.* subjects with parental education year 7-9; [e] $P < 0.01$; [f] $P < 0.001$ *vs.* subjects with parental education year 10-12 by one-Way ANOVA with Tukey's multiple comparison test . [g] *P* value by Pearson Chi-square test.

Table S3. Association between low educational status and SBP

	Model 1		Model 2		Model 3		Model 4	
	Coefficients (95%CIs)	*P* value	Coefficients (95%CIs)	*P* value	Coefficients (95%CIs)	*P* value	Coefficients (95%CIs)	*P* value
Education years ≤ 12	7.952 (7.471, 8.433)	< 0.001	3.316 (2.840, 3.792)	< 0.001	2.956 (2.487, 3.424)	< 0.001	2.938 (2.469, 3.408)	< 0.001
Age (*per* 1 year)			0.396 (0.383, 0.410)	< 0.001	0.364 (0.349, 0.379)	< 0.001	0.367 (0.349, 0.386)	< 0.001
Female sex			-4.393 (-4.819, -3.966)	< 0.001	-5.123 (-5.702, -4.544)	< 0.001	-5.088 (-5.667, -4.509)	< 0.001
High waist circumference					2.566 (1.992, 3.141)	< 0.001	2.544 (1.970, 3.118)	< 0.001
High body mass index					1.875 (1.591, 2.160)	< 0.001	1.862 (1.577, 2.147)	< 0.001
Diabetes					0.695 (0.017, 1.373)	0.045	0.517 (-0.163, 1.197)	0.136
Dyslipidemia					1.281 (0.685, 1.876)	< 0.001	1.273 (0.678, 1.869)	< 0.001
Coronary artery disease					0.225 (-1.060, 1.510)	0.731	0.187 (-1.097, 1.471)	0.775
Stroke					0.769 (-0.658, 2.195)	0.291	0.714 (-0.711, 2.140)	0.326
Anemia					-1.278 (-2.046, -0.510)	0.001	-1.339 (-1.960, -0.806)	< 0.001
Smoking					-1.336 (-1.913, -0.758)	< 0.001	-1.383 (-1.960, -0.806)	< 0.001
eGFR (mL/min./1.73m^2)							0.002 (-0.013, 0.016)	0.824
Urine protein ≥ 1+							2.310 (1.532, 3.089)	< 0.001

Note: Model 1, unadjusted. Model 2, adjusted for age and sex. Model 3, model 2 + adjusted for co-morbidities (high body mass index, high waist circumference, diabetes, dyslipidemia, coronary artery disease, stroke, and history of smoking). Model 4, model 3 + adjusted for eGFR and proteinuria). Abbreviations: CI, confidence interval; HTN, hypertension; SBP, systolic blood pressure. Abbreviations: CI, confidence interval; eGFR, estimated glomerular filtration rate.

Table S4. Association between low educational status and prevalent HTN

	Model 1		Model 2		Model 3		Model 4	
	Odds ratio (95%CI)	*P* value	Odds ratio (95%CI)	*P* value	Odds ratio (95%CI)	*P* value	Odds ratio (95%CI)	*P* value
Education years ≤ 12	2.849 (2.651, 3.061)	< 0.001	1.312 (1.205, 1.429)	< 0.001	1.203 (1.100, 1.317)	< 0.001	1.211 (1.107, 1.326)	< 0.001
Age (*per* 1 year)			1.075 (1.072, 1.078)	< 0.001	1.068 (1.065, 1.071)	< 0.001	1.065 (1.061 1.069)	< 0.001
Female sex			0.644 (0.600, 0.692)	< 0.001	0.590 (0.528, 0.659)	< 0.001	0.589 (0.528, 0.658)	< 0.001
High waist circumference					1.562 (1.421, 1.718)	< 0.001	1.564 (1.421, 1.720)	< 0.001
High body mass index					2.016 (1.834, 2.216)	< 0.001	1.985 (1.805, 2.183)	< 0.001
Diabetes					1.707 (1.538, 1.895)	< 0.001	1.657 (1.482, 1.841)	< 0.001
Dyslipidemia					2.130 (1.944, 2.333)	< 0.001	2.121 (1.936, 2.324)	< 0.001
Coronary artery disease					1.083 (0.886, 1.325)	0.435	1.069 (0.837, 1.307)	0.519
Stroke					1.940 (1.529, 2.462)	<0.001	1.919 (1.512, 2.437)	< 0.001
Anemia					1.049 (0.915, 1.203)	0.490	1.015 (0.884, 1.165)	0.835
Smoking					1.005 (0.903, 1.119)	0.926	0.998 (0.896, 1.111)	0.970
eGFR (mL/min./1.73m^2)							0.996 (0.993, 0.999)	0.003
Urine protein ≥ 1+							1.545 (1.347, 1.773)	< 0.001

Note: Model 1, unadjusted. Model 2, adjusted for age and sex. Model 3, model 2 + adjusted for co-morbidities (high body mass index, high waist circumference, diabetes, dyslipidemia, coronary artery disease, stroke, and history of smoking). Model 4, model 3 + adjusted for eGFR and proteinuria). Abbreviations: CI, confidence interval; HTN, hypertension; SBP, systolic blood pressure. Abbreviations: CI, confidence interval; eGFR, estimated glomerular filtration rate.

Table S5. Baseline characteristics of study subjects by parental educational status

	Parental education years				*P* value
	≤ 6	7-9	10-12	> 12	
Numbers	18522	5064	7193	4405	
Age (years)	57.957 ± 13.321	45.469 ± 13.099	38.918 ± 13.403	38.144 ± 14.400	< 0.001
Female (%)	10082 (54.4)	2787 (55.0)	3935 (54.7)	2353 (53.4)	0.425
Urban residence (%)	8645 (46.7)	2715 (53.6)	4139 (57.5)	2928 (66.5)	< 0.001
Education years (%)					< 0.001
≤ 6	6804 (36.7)	353 (7.0)	256 (3.6)	56 (1.3)	
7-9	2947 (15.9)	428 (8.5)	297 (4.1)	78 (1.8)	
10-12	5466 (29.5)	2219 (43.8)	2824 (39.3)	1507 (34.2)	
> 12	3305 (17.8)	2064 (40.8)	3816 (53.1)	2764 (62.7)	
Urine chemistry					
Random urine Na^+ (mEq/L)	123.947 ± 49.350	119.815 ± 51.811	117.666 ± 52.157	115.392 ± 51.707	< 0.001
Random urine Cr (mg/dL)	132.636 ± 73.792	154.261 ± 83.337	173.136 ± 92.739	179.748 ± 96.673	< 0.001
Random urine Na^+/Cr	1.226 ± 0.823	1.003 ± 0.670	0.887 ± 0.643	0.832 ± 0.574	< 0.001
Estimated 24-h urine Na^+ (g)	8.449 ± 2.087	8.099 ± 2.050	7.797 ± 2.039	7.630 ± 1.984	< 0.001
Systolic blood pressure (mmHg)	122.209 ± 17.520	115.673 ± 15.915	113.058 ± 14.449	112.186 ± 14.429	< 0.001
Diastolic blood pressure (mmHg)	75.736 ± 10.302	75.524 ± 10.558	74.583 ± 10.288	73.960 ± 9.862	< 0.001
Waist circumference (cm)	83.119 ± 9.285	81.191 ± 10.034	80.007 ± 10.593	79.045 ± 10.726	< 0.001
Body mass index (kg/m^2)	24.016 ± 3.205	23.811 ± 3.462	23.510 ± 3.693	23.169 ± 3.608	< 0.001
eGFR (mL/min./1.73m^2)	86.846 ± 16.914	95.674 ± 17.149	99.613 ± 17.249	99.319 ± 17.864	< 0.001
Urine protein ≥ 1+ (%)					
Co-morbidities					
Hypertension (%)	7354 (39.7)	1137 (22.5)	1129 (15.7)	648 (14.7)	< 0.001
Diabetes (%)	2678 (14.8)	421 (8.5)	365 (5.2)	225 (5.2)	< 0.001
Dyslipidemia (%)	3113 (30.4)	584 (21.2)	515 (12.6)	349 (12.8)	< 0.001
Coronary artery disease (%)	684 (7.3)	72 (2.8)	72 (1.8)	39 (1.5)	< 0.001
Stroke (%)	534 (5.7)	56 (2.2)	50 (1.3)	29 (1.1)	< 0.001
Anemia (%)	1739 (9.4)	423 (8.4)	500 (7.0)	321 (7.3)	< 0.001
History of smoking (%)	7319 (39.7)	2068 (40.9)	2798 (39.0)	1585 (36.0)	< 0.001

Note: Note: Values for categorical variables are given as number (percentage); values for continuous variables, as mean ± standard deviation. P value by one-way analysis of variance and χ2 test for continuous and categorical variables, respectively. Abbreviation: eGFR, estimated glomerular filtration rate.

Table S6. Association between parental educational attainment and SBP

	Model 1		Model 2		Model 3		Model 4		Model 5	
	Coefficients (95%CIs)	*P* value	Coefficients (95%CIs)	*P* value	Coefficients (95%CIs)	*P* value	Coefficients (95%CIs)	*P* value	Coefficients (95%CIs)	*P* value
Parental education years ≤ 12	6.553 (5.867, 7.240)	< 0.001	0.690 (0.037, 1.342)	0.038	0.400 (-0.239, 1.039)	0.220	0.401 (-0.238, 1.041)	0.218	-0.086 (-0.727, 0.556)	0.764
Age (*per* 1 year)			0.422 (0.408, 0.436)	< 0.001	0.384 (0.369, 0.399)	< 0.001	0.393 (0.374, 0.412)	< 0.001	0.364 (0.345, 0.383)	< 0.001
Female sex			-4.514 (-4.954, -4.074)	< 0.001	-5.063 (-5.567,-4.470)	< 0.001	-5.020 (-5.613, -4.426)	< 0.001	-5.302 (-5.895, -4.709)	< 0.001
High waist circumference					2.497 (1.901, 3.092)	< 0.001	2.467 (1.871, 3.063)	< 0.001	2.360 (1.767, 2.953)	< 0.001
High body mass index					1.998 (1.702, 2.293)	< 0.001	1.998 (-0.054, 1.382)	< 0.001	1.981 (1.686, 2.276)	< 0.001
Diabetes					0.822 (0.107, 1.538)	0.024	0.664 (-0.054, 1.382)	< 0.001	0.497 (-0.219, 1.212)	0.173
Dyslipidemia					1.619 (0.993, 2.244)	< 0.001	1.613 (0.988, 2.238)	< 0.001	1.540 (0.917, 2.163)	< 0.001
Coronary artery disease					0.246 (-1.123, 1.614)	0.725	0.228 (-1.139, 1.595)	0.744	0.115 (-1.247, 1.477)	0.868
Stroke					1.789 (0.247, 3.331)	0.023	1.755 (0.213, 3.297)	0.026	1.506 (-0.029, 3.042)	0.055
Anemia					-1.809 (-2.615, -1.003)	< 0.001	-1.828 (-2.635, -1.022)	< 0.001	-1.842 (-2.646, -1.039)	< 0.001
Smoking					-1.270 (-1.864, -0.675)	< 0.001	-1.319 (-1.914, -0.725)	< 0.001	-1.402 (-1.994, -0.810)	< 0.001
eGFR (mL/min./1.73m^2)							0.010 (-0.005, 0.025)	0.173	0.004 (-0.011, 0.019)	0.605
Urine protein ≥ 1+							2.109 (1.299, 2.919)	<0.001	2.030 (1.223, 2.837)	< 0.001
Education years ≤ 12									2.938 (2.460, 3.416)	< 0.001

Note: Model 1, unadjusted. Model 2, adjusted for age and sex. Model 3, model 2 + adjusted for co-morbidities (high body mass index, high waist circumference, diabetes, dyslipidemia, coronary artery disease, stroke, and history of smoking). Model 4, model 3 + adjusted for eGFR and proteinuria). Model 5, model 4 + adjusted for educational status of individual subjects. Abbreviations: eGFR, estimated glomerular filtration rate.

Table S7. Association between parental educational attainment and prevalent HTN

	Model 1		Model 2		Model 3		Model 4		Model 5	
	Odds ratio (95%CI)	*P* value	Odds ratio (95%CI)	*P* value	Odds ratio (95%CI)	*P* value	Odds ratio (95%CI)	*P* value	Odds ratio (95%CI)	*P* value
Parental education years ≤ 12	2.638 (2.419, 2.877)	< 0.001	1.138 (1.032, 1.254)	< 0.001	1.149 (1.002, 1.316)	0.046	1.157 (1.010, 1.326)	0.036	1.104 (0.961, 1.269)	0.161
Age (*per* 1 year)			1.079 (1.077, 1.081)	< 0.001	1.070 (1.066, 1.073)	< 0.001	1.067 (1.064, 1.071)	< 0.001	1.065 (1.061, 1.069)	< 0.001
Female sex			0.675 (0.641, 0.711)	< 0.001	0.598 (0.533, 0.670)	< 0.001	0.598 (0.534, 0.674)	< 0.001	0.580 (0.5174, 0.651)	< 0.001
High waist circumference					1.566 (1.417, 1.730)	< 0.001	1.569 (1.419, 1.743)	< 0.001	1.565 (1.416, 1.730)	< 0.001
High body mass index					2.045 (1.852, 2.259)	< 0.001	2.017 (1.825, 2.229)	< 0.001	2.010 (1.819, 2.221)	< 0.001
Diabetes					1.710 (1.531, 1.910)	< 0.001	1.663 (1.488, 1.859)	< 0.001	1.653 (1.478, 1.847)	< 0.001
Dyslipidemia					2.192 (1.991, 2.413)	< 0.001	2.183 (.983, 2.404)	< 0.001	2.184 (1.983, 2.404)	< 0.001
Coronary artery disease					0.998 (0.807, 1.234)	0.984	0.987 (0.798, 1.222)	0.908	0.984 (0.795, 1.218)	0.883
Stroke					1.971 (1.523, 2.550)	< 0.001	1.952 (1.508, 2.527)	< 0.001	1.928 (1.489, 2.496)	< 0.001
Anemia					1.005 (0.868, 1.164)	0.943	0.977 (0.843, 1.133)	0.759	0.974 (0.840, 1.129)	0.728
Smoking					1.002 (0.896, 1.121)	0.969	0.995 (0.890, 1.113)	0.993	0.984 (0.880, 1.101)	0.783
eGFR (mL/min./1.73m^2)							0.997 (0.994, 1.000)	0.023	0.996 (0.994, 0.999)	0.013
Urine protein ≥ 1+							1.517 (1.313, 1.753)	< 0.001	1.511 (1.307, 1.746)	< 0.001
Education years ≤ 12									1.177 (1.071, 1.293)	0.001

Note: Model 1, unadjusted. Model 2, adjusted for age and sex. Model 3, model 2 + adjusted for co-morbidities (high body mass index, high waist circumference, diabetes, dyslipidemia, coronary artery disease, stroke, and history of smoking). Model 4, model 3 + adjusted for eGFR and proteinuria). Model 5, model 4 + adjusted for educational status of individual subjects. Abbreviations: CI, confidence interval; eGFR, estimated glomerular filtration rate.

Table S8. Association between parental educational status and random urine Na^+/Cr

	Model 1		Model 2		Model 3		Model 4		Model 5	
	Coefficients (95%CIs)	*P* value	Coefficients (95%CIs)	*P* value	Coefficients (95%CIs)	*P* value	Coefficients (95%CIs)	*P* value	Coefficients (95%CIs)	*P* value
Parental education years ≤ 12	0.243 (0.214, 0.271)	< 0.001	0.038 (0.010, 0.066)	0.007	0.033 (0.006, 0.061)	0.017	0.033 (0.006, 0.061)	0.017	0.019 (-0.008, 0.047)	0.173
Age (*per* 1 year)			0.015 (0.014, 0.015)	< 0.001	0.013 (0.013, 0.014)	< 0.001	0.015 (0.015, 0.016)	< 0.001	0.015 (0.014, 0.015)	< 0.001
Female sex			0.228 (0.209, 0.247)	<0.001	0.185 (0.159, 0.211)	< 0.001	0.187 (0.162, 0.213)	< 0.001	0.179 (0.153, 0.204)	< 0.001
High waist circumference					0.043 (0.017, 0.069)	0.001	0.039 (0.014, 0.065)	0.003	0.036 (0.011, 0.062)	0.005
High body mass index					-0.007 (-0.020, 0.006)	0.289	0.000 (-0.013, 0.012)	0.950	-0.001 (-0.013, 0.012)	0.919
Hypertension					0.039 (0.015, 0.063)	0.001	0.055 (0.032, 0.079)	< 0.001	0.051 (0.028, 0.075)	< 0.001
Diabetes					0.019 (-0.012, 0.051)	0.230	0.047 (0.017, 0.078)	0.003	0.043 (0.012, 0.074)	0.043
Dyslipidemia					0.034 (0.007, 0.062)	0.015	0.038 (0.011, 0.065)	0.006	0.036 (0.009, 0.063)	0.008
Coronary artery disease					0.005 (-0.055, 0.064)	0.874	0.013 (-0.046, 0.072)	0.663	0.010 (-0.049, 0.068)	0.741
Stroke					-0.059 (-0.126, 0.008)	0.086	-0.045 (-0.111, 0.021)	0.186	-0.051 (-0.117, 0.015)	0.127
Anemia					0.065 (0.030, 0.100)	< 0.001	0.085 (0.051, 0.120)	< 0.001	0.085 (0.050, 0.119)	< 0.001
Smoking					-0.053 (-0.079, -0.028)	< 0.001	-0.048 (-0.073, -0.022)	< 0.001	-0.050 (-0.076, -0.025)	< 0.001
eGFR (mL/min./1.73m^2)							0.003 (0.003, 0.004)	< 0.001	0.003 (0.003, 0.004)	< 0.001
Urine protein ≥ 1+							-0.377 (-0.412, -0.343)	< 0.001	-0.379 (-0.414, -0.345)	< 0.001
Education years ≤ 12									0.086 (0.066, 0.107)	< 0.001

Note: Model 1, unadjusted. Model 2, adjusted for age and sex. Model 3, model 2 + adjusted for co-morbidities (high body mass index, high waist circumference, diabetes, dyslipidemia, coronary artery disease, stroke, and history of smoking). Model 4, model 3 + adjusted for eGFR and proteinuria). Model 5, model 4 + adjusted for educational status of individual subjects. Abbreviations: CI, confidence interval; eGFR, estimated glomerular filtration rate.
